# Analysis of depression prevalence and associated influencing factors in maintenance hemodialysis patients

**DOI:** 10.3389/fpsyt.2025.1682681

**Published:** 2025-11-28

**Authors:** Li Cao, Yongming Wu, Ruibo Deng, Xinwen Zhang, Hehua Li, Jiamin Li, Chenyu Liu, Ziyun Zhang, Lin Zhu, Yuanyuan Huang

**Affiliations:** 1 Yancheng First Hospital, Affiliated Hospital of Nanjing University Medical School, Yancheng, China; 2 The First People’s Hospital of Yancheng, Yancheng, China; 3 Nanjing Medical University, Nanjing, China; 4 Guangzhou University of Chinese Medicine, Guangzhou, China; 5 Jiangsu Normal University, Xuzhou, China; 6 The Affiliated Brain Hospital, Guangzhou Medical University, Guangzhou, China; 7 Baiyun District People’s Hospital of Guangzhou, Guangzhou, China; 8 Guangdong Engineering Technology Research Center for Translational Medicine of Mental Disorders, Guangzhou, China

**Keywords:** maintenance hemodialysis, depression, self-care ability, influencing factors, Barthel Index

## Abstract

**Background:**

Hemodialysis is a common and effective treatment method for end-stage renal disease, but it is associated with adverse reactions, which may impair patients’ daily functioning and contribute to psychological burden. This study aims to investigate the prevalence of depression in maintenance hemodialysis (MHD) patients and its correlation with self-care ability.

**Methods:**

A total of 152 MHD patients were enrolled in this study. Depression was assessed using the Self-Rating Depression Scale (SDS), with SDS scores ≥ 53 classified as the depression group and < 53 as the non-depression group. Self-care ability was assessed using the Barthel Index (BI). General clinical data and self-care ability were compared between the two groups. The correlation between depression and self-care ability (including basic mobility, spontaneous excretion, physical activity, and total scores) was analyzed in the depression group.

**Results:**

Among 152 patients, 82 exhibited depressive symptoms (39 mild, 35 moderate, and 8 severe). The depression and non-depression groups showed statistically significant differences in occupation and education level (*p* < 0.05), but no significant differences in gender, employment status, marital status, comorbidities, or age (*p* > 0.05). The depression group had significantly lower scores in basic mobility, spontaneous excretion, physical activity, and BI total scores (*p* < 0.05). These self-care dimensions were negatively correlated with SDS scores in the depression group.

**Conclusion:**

The prevalence of depression is high among MHD patients, and depressive symptoms are closely associated with impaired self-care ability.

## 1 Introduction

End-stage renal disease (ESRD), as the final stage of chronic kidney disease (CKD), has become a global public health challenge. It is characterized by irreversible renal failure and a high incidence of multi-system complications, including diabetes, hypertension, and cardiovascular diseases (1, 2). Epidemiological studies have shown that the global prevalence of ESRD is increasing at a rate of 10% to 15% annually (3–5), and this phenomenon is particularly prominent in developing countries. As a populous country in the world, China is facing an increasingly severe burden of ESRD under the dual influence of the accelerating aging process of the population and the rising prevalence of metabolic diseases such as diabetes and hypertension. Hemodialysis (HD), as the main renal replacement therapy for ESRD (6, 7), can effectively correct uremic symptoms such as azotemia, fluid and electrolyte imbalances, and acid-base imbalance by establishing extracorporeal circulation (8). Although this technology can significantly prolong the survival period of patients, its clinical application still has obvious limitations: complications such as hypotension and cardiovascular events are prone to occur during the treatment process, which directly affect the treatment tolerance and long-term prognosis of patients. This characteristic of coexisting efficacy and risk highlights the necessity of optimizing treatment strategies.

It is worth noting that hemodialysis patients are confronted with multi-dimensional health challenges. Clinical observations show that: At the physiological level, patients continuously bear the burden of uremia-related symptoms (9), At the psychological level, mental disorders such as depression are highly prevalent. Research shows that approximately 55.1% of hemodialysis patients have depressive symptoms, which are significantly associated with reduced treatment compliance and decreased quality of life (10), At the socio-economic level, the high cost of dialysis treatment has imposed a heavy burden on the medical system (11). It is particularly important to note that cognitive distortions caused by depression may intensify patients’ negative attitudes towards treatment, thereby creating a vicious cycle of symptom aggravation, reduced treatment compliance, and deteriorating prognosis, and even increasing the risk of suicide (12). However, existing research still has limitations in the analysis of the specific influence mechanism of uremia and the control of socio-economic factors.

Activities of daily living (ADL), especially self-care ability, as a core indicator for evaluating the quality of life of patients (13), is generally impaired in the hemodialysis population (14). Studies have shown that hemodialysis treatment can significantly reduce the ADL level of patients, leading to an increase in their dependence on care (15). This decline in self-care ability not only directly affects the quality of life of patients, but may also further deteriorate the overall health condition by aggravating depressive tendencies (16). Therefore, in-depth exploration of the interaction mechanism between self-care ability and depressive symptoms is of great clinical significance for improving the comprehensive prognosis of ESRD patients.

Therefore, a thorough understanding of hemodialysis patients’ mental health and its influencing factors is essential to implementing targeted interventions that improve these outcomes. This study hypothesizes a strong correlation between the self-care ability of hemodialysis patients and their depressive tendencies. Through systematic investigation, it aims to analyze the prevalence of depressive symptoms and relevant influencing factors among hemodialysis patients, thereby providing a scientific basis for clinical psychological interventions.

## 2 Methods

### 2.1 Participants

A total of 152 MHD patients from April 2025 to June 2025 were randomly recruited into this cross-sectional study. The study was approved by the Ethics Committee of Yancheng First People’s Hospital (Ethics Approval No.2025-K-147), and all patients provided written informed consent. Inclusion criteria: 1) Age ≥18 years; 2) Diagnosed with ESRD based on the diagnostic criteria for chronic kidney disease stage 5; 3) Currently undergoing MHD for more than 1 year; 4) Ability to understand and complete the questionnaires (independently or with assistance). Exclusion criteria: 1) Mental disorders diagnosed according to DSM-5 prior to MHD treatment; 2) Drug or alcohol dependence; 3) Use of antidepressant medications within the last 3 months; 4) Comorbid currently severe or unstable high blood pressure/diabetes; 5) Serious comorbidities, for instance, active malignancies or unstable cardiac disease.

### 2.2 General information and clinical symptom assessment

General demographic and clinical information were collected, including gender, age, years of education, and comorbidities such as hypertension, heart disease, and thyroid disorders. Additionally, information on the patients’ level of family care and history of marriage and childbearing was collected.

Depressive symptoms were assessed using the Self-Rating Depression Scale (SDS). The SDS consists of 20 items (including feelings of depression and crying tendencies), with each item scored from 1 to 4 points. The raw score was multiplied by 1.25 to convert it into a percentage-based score. A total score ≥ 53 indicated depressive symptoms, with scores of 53-62, 63-72, and ≥ 73 representing mild, moderate, and severe depression, respectively. Patients with SDS scores ≥ 53 were classified into the depression group, while those with scores < 53 were assigned to the non-depression group (17).

Patients’ self-care ability was assessed using the Barthel Index (BI) (18). The BI scale comprises 10 items (including feeding and bathing), with each item scored from 0 to 15 points. Higher total scores indicate better self-care ability (19). In accordance with the collective clinical practice of our research members and the affiliated hospital staff, the items were grouped into three dimensions based on their content: basic activities, spontaneous excretion, and physical mobility (with 3–4 items per group). Higher subgroup scores reflect better self-care ability in that particular dimension. The BI Alpha credibility score is 0.914, and the SDS Alpha credibility score is 0.795, both meeting the research requirements.

To ensure data reliability, all scales were completed by participants with the assistance of trained research team members who underwent standardized training.

### 2.3 Statistical analysis

All statistical analyses were performed using SPSS 27.0 software. The Kolmogorov-Smirnov test was used to assess the normality of continuous variables, and the chi-square (χ²) test was used for intergroup comparisons of categorical variables. Normally distributed data were presented as mean ± standard deviation (x ± s). The continuous variables were found to follow a normal distribution and were compared between groups using independent samples t-test. Since the data followed a normal distribution, Pearson correlation analysis was employed in this study to examine the correlations between depressive symptoms and demographic characteristics as well as self-care ability. The significance level was set at α=0.05.

## 3 Results

### 3.1 Demographic data and depression incidence

A total of 152 participants were included, comprising 98 males and 54 females, with ages ranging from 18 to 84 years (mean age: 53.13 ± 13.13). Depressive symptoms were detected in 82 cases (54%), including 39 mild (47.56%), 35 moderate (42.68%), and 8 severe cases (9.76%).

### 3.2 Intergroup comparison of baseline characteristics

The intergroup comparison revealed that years of education and occupation showed statistically significant differences between the depression and non-depression groups (*p* < 0.05). However, no significant differences were observed between the two groups in terms of age, gender, or place of residence (*p* > 0.05), as presented in **Table 1**.

**Table 1:** Comparison of baseline characteristics between the two groups.

Variables		Non-depressive group (n=70)	Depression group (n=82)	*t*	χ^2^	*p*
Age		54.88 ± 13.79	52.39 ± 12.28	-1.168		0.245
Years of education		1.91 ± 1.13	1.49 ± 0.78	-2.675		0.009
Gender	Man	52(63.4%)	46(65.7%)		0.087	0.768
	Woman	30(36.6%)	24(34.3%)			
Complications	Yes	62(75.6%)	36(51.4%)		0.253	0.615
	No	20(24.4%)	34(48.6%)			
Place of residence	Countryside	26(31.7%)	20(28.6%)		0.176	0.675
	Town	56(68.3%)	50(71.4%)			

Comorbidities include hypertension, heart disease, anemia, thyroid disorders, etc.

### 3.3 Comparative analysis of self-care capacity between the two groups

The depressive group demonstrated statistically significant lower scores in BI, physical mobility, and overall self-care capacity compared to controls (all *p* < 0.05; **Table 2**).

**Table 2:** Comparison of self-care indices between the two groups.

Scales	Non-depressive group(n=70)	Depression group(n=82)	*t*	*p*
BI Total Score	59.43 ± 2.49	55.91 ± 11.47	2.512	0.013
Foundational Activities	29.71 ± 1.45	27.2 ± 6.72	3.076	0.002
Spontaneous Excretion	29.71 ± 1.68	28.6 ± 5.84	1.545	0.124
Physical Exercise	38.79 ± 4.21	34.7 ± 9.44	3.351	0.001
SDS Standard Score	42.95 ± 5.85	62.52 ± 6.69	-19.036	<0.001

### 3.4 Correlation between depressive symptoms and demographic characteristics/self-care ability

**Figure 1:**
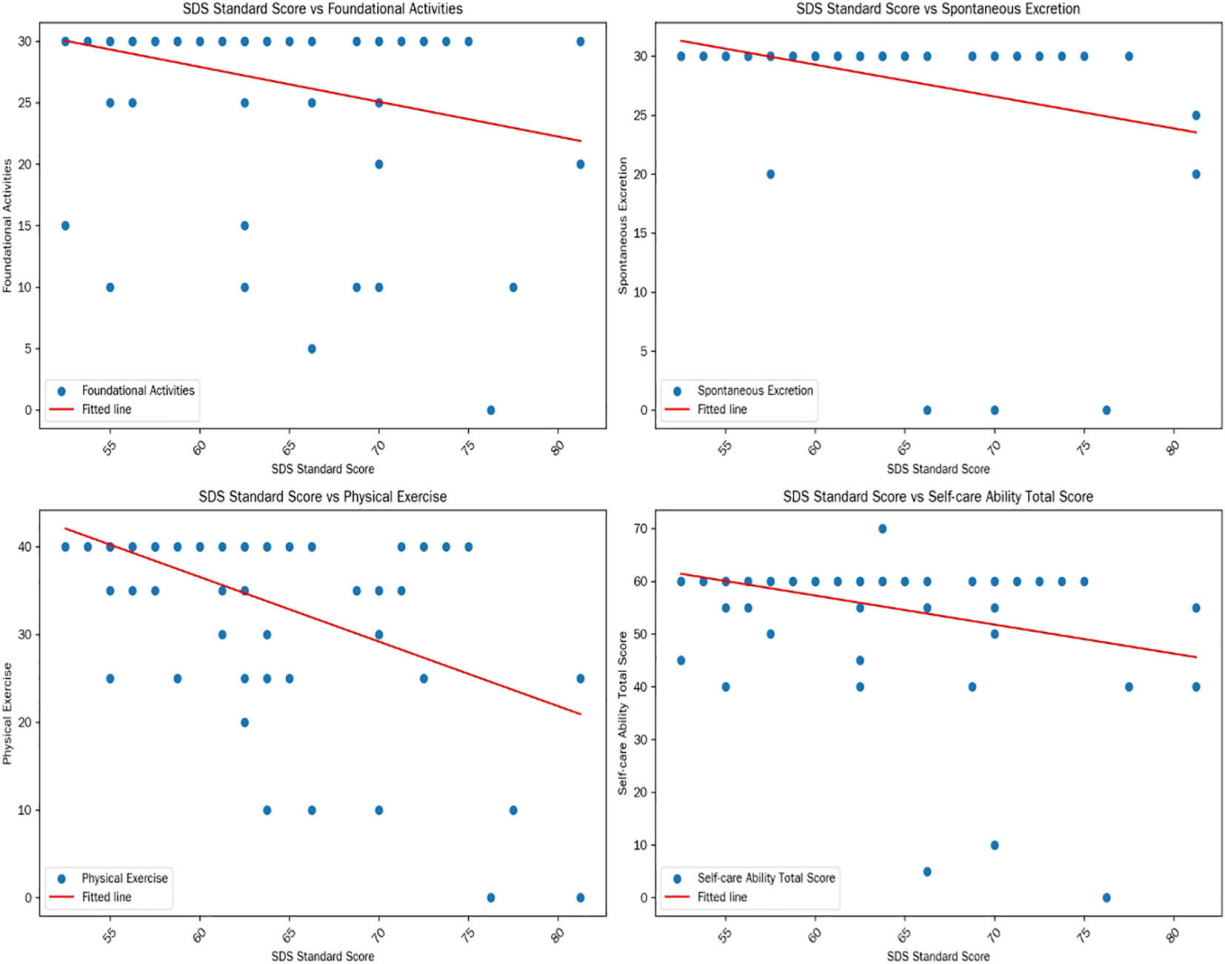
Correlation between self-care ability and SDS scores in the depression group.

In the depression group, both educational attainment and self-care ability scores demonstrated statistically significant negative correlations with SDS scores (**Table 3**). Furthermore, significant inverse associations were observed between SDS standard scores and performance in basic activities, spontaneous excretion, physical mobility, as well as total self-care capacity (all *p* < 0.05, **Table 4** and **Figure 1**).

**Table 3:** Correlations between baseline characteristics and SDS scores in the depression group.

Constituencies	*r*	*p*
Age (years)	0.136	0.096
Years of education (years)	-0.328	<0.001
Gender (Man/Woman)	0.127	0.119
Place of residence (Countryside/Town)	-0.068	0.410
The Level of Care of the Family (Concerned/Average/Not concerned)	-0.049	0.547
History of Marriage and Childbearing (Married with children/Married without children/Unmarried and childless)	-0.019	0.816
Self-care Ability Total Score (Scores)	-0.321	0.003

**Table 4:** Correlation between self-care ability and SDS scores in the depression group.

Constituencies	SDS Standard score	
	*r*	*p*
Foundational Activities	-0.282	0.010
Spontaneous Excretion	-0.309	0.005
Physical Exercise	-0.522	<0.001
Self-care Ability Total Score	-0.321	0.003

The total BI score equaled the sum of the scores from the remaining three groups.

## 4 Discussion

This study employed the SDS and BI to investigate the prevalence of depressive symptoms and their correlation with self-care ability among MHD patients. Our key findings include: 1) The prevalence of depressive symptoms is high in patients with maintenance hemodialysis; 2) the depressed group showed significantly lower scores in basic activities, spontaneous excretion, physical mobility, and total BI scores compared to the non-depressed group; 3) these BI subscale scores and total scores demonstrated negative correlations with SDS standardized scores in the depressed group; 4) an association between depressive tendencies and educational attainment. Our results primarily indicate that depressive tendencies in MHD patients are closely associated with self-care capacity, where lower self-care ability is associated with higher depressive symptom burden. These findings provide valuable references for the diagnosis and treatment of psychological comorbidities in hemodialysis patients.

This study found that 53.95% of MHD patients exhibited depressive symptoms such as low mood, poor nighttime sleep quality, and unexplained fatigue, which is consistent with previous research findings (20, 21). MHD patients have a high prevalence of depressive disorders, and the mortality risk for patients with depressive disorders is higher than that of common MHD comorbidities such as anemia and frequent intradialytic hypotension, which are often the focus of clinical attention (22). Patients with chronic renal failure undergoing hemodialysis often lack sufficient knowledge about the etiology, pathogenesis, prognosis, and complications of their disease. Additionally, the high cost and prolonged treatment duration of hemodialysis can easily lead to negative emotions such as anxiety, depression, and illness-related uncertainty during the treatment process (23). This psychological burden may reduce patient compliance and self-care ability, diminish treatment effectiveness, and severely impact their quality of life (24). Such outcomes may result in poor disease prognosis, increase the financial burden on patients’ families, and even exacerbate the societal burden of healthcare maintenance.

This study found that hemodialysis patients with depressive tendencies may exhibit weaker self-care abilities, which is consistent with previous research findings (25). At the physiological level, the impact of depressive tendencies on self-care ability in hemodialysis patients is primarily reflected in neurochemical mechanisms and brain functional changes. First, neurotransmitter system dysregulation is the core pathological basis—deficient serotonin (5-HT) secretion significantly reduces motivation and emotional regulation, while dopaminergic system dysfunction directly affects the reward circuit, leading to loss of interest and pleasure in daily activities (26). This dual neurotransmitter imbalance contributes to persistent fatigue and initiation deficits, making even basic self-care tasks challenging. Second, neuroimaging studies confirm that chronic depression leads to reduced metabolic activity and gray matter volume loss in the prefrontal cortex (particularly the dorsolateral prefrontal cortex), a key region for executive function. Impairment in this area directly affects decision-making, task planning, and behavioral regulation (27). These physiological changes often precede behavioral manifestations, serving as early warning signs of declining self-care ability. At the psychological level, depressive patients’ negative cognitions (e.g., self-denial and pessimistic expectations) significantly reduce their self-care motivation, while persistent emotional exhaustion makes even basic activities feel overwhelming, creating psychological inertia. Cognitive distortions and emotional depletion reinforce each other, forming a vicious cycle that hinders self-care behaviors (28). This state may even diminish patients’ adherence and responsiveness to rehabilitation interventions. At the behavioral level, hemodialysis patients with depressive tendencies gradually reduce self-care activities, developing avoidant behaviors such as skipping medical follow-ups and neglecting personal hygiene. This activity reduction and functional decline reinforce each other, ultimately forming a “reduced activity → functional deterioration → increased avoidance” vicious cycle (29). This behavioral regression often begins with avoidance of complex tasks and eventually extends to abandoning basic daily activities. At the environmental level, depressive symptoms in hemodialysis patients are closely linked to inadequate social support systems. Societal stigma and discriminatory attitudes toward mental illness further create barriers, discouraging patients from seeking help even when aware of their psychological distress, ultimately worsening depressive symptoms and accelerating physical functional decline (30).These factors interact dynamically, making it difficult for patients to maintain basic self-care. Therefore, comprehensive interventions—including pharmacotherapy, behavioral activation, and socio-environmental adjustments—are necessary (31).

In our sample, we examined the relationship between self-care ability and depressive symptoms. The results showed a negative correlation between patients’ self-care ability and depressive symptoms. A possible explanation is that higher self-care ability enables individuals to independently meet their basic daily needs without relying on others, enhancing autonomy and independence (32). This may contribute to personal growth and improved quality of life, thereby alleviating depressive symptoms. Furthermore, based on the scale’s characteristics, we categorized self-care ability into three dimensions—basic activities, spontaneous excretion, and physical mobility—to reflect patients’ self-care levels across different aspects. The results showed that scores in all three dimensions were negatively correlated with depressive symptoms.

A higher “basic activities” score reflects greater independence in daily tasks, fostering a sense of achievement. Studies indicate this helps patients recognize their capabilities despite illness, boosting self-confidence and reducing depression risk through positive reinforcement (33). Task completion enhances life control, countering depressive helplessness (34). Bandura’s self-efficacy theory notes achieving small goals reinforces “I can do it,” improving mood (35), while task focus diverts from negative thoughts, akin to mindfulness (36), reducing rumination (37). A higher “spontaneous excretion” score indicates better urinary/defecatory control and toileting independence. Autonomy in excretion is a fundamental physiological control; its loss can trigger existential anxiety (38) and a “meaninglessness-depression” cycle (39). Managing elimination upholds privacy and dignity, reinforcing body management confidence (40). Incontinence concerns, especially in social settings, heighten anxiety. Roy’s Adaptation Model links toileting deficits to body image-role maladaptation and social withdrawal (41), while excretory control alleviates this anxiety (42, 43). Analysis suggests that depressive tendencies in MHD patients show little correlation with autonomous excretion ability. This is likely because most patients do not face significant excretion-related issues. Regarding urination, the vast majority of MHD patients are anuric or oliguric. Fluid balance is managed through dialysis rather than renal function, so the act of “urination” is largely absent from their daily lives. As a result, the emotional impact of losing a function that is already minimal is negligible. As for defecation, it is typically controlled through established medications (e.g., laxatives) and nursing protocols. This predictability and controllability greatly reduce uncertainty and shame. In contrast, psychological issues such as depressive mood and altered self-image lack similarly direct and effective interventions. A higher “physical mobility” score indicates better independent movement ability. Physical activity has been shown to alleviate depression and improve daily function (44). It activates the brain’s reward system, increasing dopamine (enhancing motivation) and endorphins (reducing pain and anxiety), thus countering depressive anhedonia (45, 46). MHD patients often face chronic stress, which elevates pro-inflammatory cytokines (e.g., IL-6, TNF-α) and impairs neuroplasticity. Physical activity mitigates neuroinflammation via muscle-derived anti-inflammatory factors like IL-10 (47). Exercise thus has a “moderate to large significant effect” on depression, and greater mobility significantly reduces depression risk in patients (48). In summary, depressive symptoms in MHD patients are closely linked to both overall self-care ability and its specific dimensions. Clinically, efforts should focus on maintaining and improving self-care capacity while providing psychological support and depression prevention for those with limited independence. This approach may help reduce depressive symptom burden, alleviate caregiving burdens, enhance patients’ life satisfaction, support adherence, and improve treatment efficacy.

Our findings also revealed a negative correlation between depressive symptoms and years of education in maintenance hemodialysis patients. Current evidence supports an overall inverse relationship between education level and depression risk, with studies showing that individuals with less than a bachelor’s degree have 1.5–2 times higher depression rates than those with bachelor’s degrees or higher (2019 data). This may be because higher education typically leads to better income, more stable employment, and improved healthcare access - all factors that can buffer against depression risk. Additionally, education enhances problem-solving skills and emotional regulation abilities; for instance, more educated individuals tend to be better at utilizing mental health resources (49). Furthermore, greater knowledge retention may facilitate patients’ use of self-management scales, improve the quality of patient-reported data collection, enhance treatment efficiency, reduce suffering, and ultimately lower depression risk (50).

This study has several limitations: (1) The sample size was limited and should be expanded in future research; (2) Although data collection was conducted one-on-one by dedicated personnel, we relied solely on self-report scales without diagnostic screening or objective indicators such as laboratory tests; (3) As a cross-sectional study, it lacks longitudinal follow-up data and cannot establish causal relationships. Future studies should incorporate larger sample sizes and objective data for prospective follow-up research to provide better references for the prevention and treatment of emotional problems in maintenance hemodialysis patients; (4) The categorization of the BI into three domains represents an approach specific to this investigation, posing potential limitations to the generalizability of the outcomes.

In conclusion, the prevalence of depressive symptoms is high in MHD patients and is associated with lower self-care ability. This study suggests that patients with limited self-care ability may require prioritized monitoring and supportive interventions.

## Data Availability

The raw data supporting the conclusions of this article will be made available by the authors, without undue reservation.
